# Source Camera Identification with a Robust Device Fingerprint: Evolution from Image-Based to Video-Based Approaches

**DOI:** 10.3390/s23177385

**Published:** 2023-08-24

**Authors:** Manisha, Chang-Tsun Li, Karunakar A. Kotegar

**Affiliations:** 1 Department of Data Science and Computer Applications, Manipal Institute of Technology, Manipal Academy of Higher Education, Manipal 576104, India; nimamanisha@gmail.com (M.); karunakar.ak@manipal.edu (K.A.K.); 2 School of Information Technology, Deakin University, Geelong 3216, Australia

**Keywords:** multimedia forensics, video forensics, source camera identification, PRNU, deep learning, convolutional neural network

## Abstract

With the increasing prevalence of digital multimedia content, the need for reliable and accurate source camera identification has become crucial in applications such as digital forensics. While effective techniques exist for identifying the source camera of images, video-based source identification presents unique challenges due to disruptive effects introduced during video processing, such as compression artifacts and pixel misalignment caused by techniques like video coding and stabilization. These effects render existing approaches, which rely on high-frequency camera fingerprints like Photo Response Non-Uniformity (PRNU), inadequate for video-based identification. To address this challenge, we propose a novel approach that builds upon the image-based source identification technique. Leveraging a global stochastic fingerprint residing in the low- and mid-frequency bands, we exploit its resilience to disruptive effects in the high-frequency bands, envisioning its potential for video-based source identification. Through comprehensive evaluation on recent smartphones dataset, we establish new benchmarks for source camera model and individual device identification, surpassing state-of-the-art techniques. While conventional image-based methods struggle in video contexts, our approach unifies image and video source identification through a single framework powered by the novel non-PRNU device-specific fingerprint. This contribution expands the existing body of knowledge in the field of multimedia forensics.

## 1. Introduction

Multimedia forensics is a field that focuses on the analysis and investigation of multimedia data such as digital images and videos to uncover media provenance and various types of manipulations, forgeries, and tampering. It plays a crucial role in ensuring the origin, authenticity, integrity, and trustworthiness of digital visual content. Techniques and algorithms developed in multimedia forensics have significantly advanced our ability to extract valuable information from images/videos, leading to breakthroughs in various applications, including source camera identification.

Source camera identification is a subfield of multimedia forensics that aims to determine the specific device that captured a given image or video [1]. This identification process relies on the extraction and analysis of unique characteristics present in the digital images, often referred to as camera-specific fingerprints. By associating an image with its source camera, investigators can establish the authenticity of an image, link it to a particular device, and potentially identify the individual responsible for capturing the image. Over the years, significant progress has been made in the development of techniques for image-based source camera identification. These techniques exploit the intrinsic imperfections in imaging sensors, such as Photo Response Non-Uniformity (PRNU), which can act as a unique identifier for each camera [2]. By analyzing the statistical properties of PRNU, researchers have successfully established correlations between image artifacts and the source camera, leading to accurate identification of the camera device [2,3,4].

Over the past decade, substantial progress has been made in source identification through the extraction of the PRNU fingerprint from images [2,3,4,5,6,7,8,9,10]. The pioneering work of Lukas et al. [2] has greatly contributed to the advancement of research in this field. Many contemporary studies estimate PRNU as the residual noise obtained by subtracting a denoised image from the original image [2,3,4,5,6,7]. Researchers have also strived to enhance the accuracy of source identification by mitigating undesired artifacts such as scene details [7] and irrelevant noise, thereby improving the quality and reliability of PRNU fingerprints derived from images [8,9,10]. Despite its status as the most promising technique in source camera identification, the PRNU fingerprint possesses inherent limitations, including susceptibility to interference by scene details [7], vulnerability to incidental image processing, and susceptibility to counter-forensic attacks [11,12,13,14,15,16]. Consequently, multimedia forensic researchers have developed diverse frameworks employing deep learning techniques to autonomously learn discriminative features that assist in forensic investigations.

In recent years, there has been a notable shift towards a data-driven approach in source camera identification, leveraging Convolutional Neural Networks (CNNs) to learn camera-specific features that map images to their respective source cameras. While significant progress has been made in utilizing deep learning techniques for camera model identification [17,18,19,20,21,22,23,24,25,26,27,28], several challenges still demand attention. One such challenge involves differentiating between devices of the same model, which is crucial for achieving precise traceability. However, individual device identification poses a greater challenge for deep learning-based methods compared to brand or model identification, as these systems automatically look beyond the PRNU fingerprint and incorporate common in-camera processing techniques shared by all devices of the same model or brand. To address this challenge, image forensic researchers have developed deeper CNN architectures with a specific focus on individual device identification [29,30,31,32,33,34,35]. Additionally, there have been attempts to combine data-driven and hand-crafted features to further enhance the accuracy of distinguishing between individual devices [31]. However, it is important to note that the current accuracy achieved in differentiating between devices of the same model falls short of meeting the requirements for forensic applications. This discrepancy stems from the possibility that the learned traits within CNNs designed for camera model identification may differ from the device-specific traits necessary for distinguishing between individual devices.

As a potential solution that overcomes the limitations associated with PRNU fingerprinting and existing data-driven techniques, our seminal work [36] has uncovered the existence of a novel fingerprint other than PRNU in digital images that is inherently unique to each device. We discovered that the new device fingerprint is location-independent, stochastic, and globally available, which resolves the spatial synchronization issue. Unlike the PRNU, which resides in the high-frequency bands, the new device fingerprint is extracted from the low- and mid-frequency bands, which resolves the fragility issue that the PRNU is unable to contend with. Our experiments on multiple datasets have shown that the new device-specific fingerprint is remarkably resistant to image alterations, such as rotation, gamma correction, and aggressive JPEG compression. This discovery opens up new avenues for source camera identification, offering a more robust and device-specific approach.

While image-based source camera identification has achieved notable success, extending these methodologies to the domain of video-based source camera identification presents additional challenges [37]. In addition to the reason that videos are of lower resolution and poorer quality than images, another possible reason is that a camera employs different algorithms in its processing pipeline for images and videos. Videos introduce temporal dependencies, motion, video stabilization, and diverse encoding formats, which significantly complicate the identification process. Analyzing video content frame by frame becomes insufficient, requiring specialized techniques to capture the temporal characteristics and variations in the source camera’s fingerprint across multiple frames. Moreover, the video frames are compressed with relatively low quality than an image through video coding. Another major problem is video stabilization, which causes the misalignment of individual pixels across frames. Thus, the estimation of PRNU from the video is much more challenging [38,39,40]. However, video-based source camera identification holds immense importance in various domains. In law enforcement, it can provide critical evidence for criminal investigations, enabling the linking of video evidence to specific cameras, aiding in suspect identification, and supporting prosecution. For example, the following scenario demonstrates the necessity of digital forensic techniques to effectively identify the source camera of videos. *“A police complaint is lodged against a person, who has allegedly installed a spy camera to capture voyeuristic videos of a person. In this scenario, linking a set of voyeuristic videos recorded on the hard disk of the spy camera to another video stored on the offender’s mobile phone, which was originally produced by the offender’s spy camera, would be a viable approach for establishing the claims”*. In this scenario, it is crucial to link the video to the offender’s device to establish the source and validate any claims against the accused. The application of source camera identification techniques in real-world criminal cases by law enforcement sectors (e.g., Guildford Crown Court, U.K. Sussex Police, and INTERPOL) has demonstrated the significance of source identification in the fight against crime. For example, in 2016, a set of voyeuristic videos in the hard disk of a spy camera were linked to another video stored in the offender’s mobile phone by Guildford Crown Court (UK) using device fingerprinting [41]. The offender pleaded guilty to fixing the spy camera. This emphasizes the need for efficient forensic techniques to trace back the videos to the camera device held by the accused to prove all claims against the accused to be true.

In our earlier work [36], we focused on the analysis and investigation of digital images, uncovering a novel fingerprint that resides in the low- and mid-frequency bands. This fingerprint exhibited inherent uniqueness to each device and showcased robustness when compared to fingerprints like PRNU, which typically reside in high-frequency bands. These characteristics of the newly discovered fingerprint offer significant potential for video-based source camera identification. This is due to the fact that compression algorithms usually affect the high-frequency bands of images to a greater extent. As a result, the new fingerprint’s location in the lower frequency bands provides increased resilience against compression artifacts commonly introduced by video coding. Additionally, the global stochastic and location independence of the new fingerprint overcomes issues caused by transformations like video stabilization, which can misalign pixels across frames. Furthermore, the fingerprint’s resistance to various image alterations and compression artifacts, including rotation, gamma correction, and aggressive JPEG compression, makes it well-suited for video analysis. Videos are often subjected to lower-quality compression compared to images, and the robustness of the new fingerprint to these artifacts ensures accurate identification even in the presence of video-specific distortions. Considering these advantageous characteristics, we hypothesize that the new device fingerprint [36] can be successfully extended to video-based source camera identification. By testing the feasibility and performance of this fingerprint in the context of videos, we strive to enhance the forensic capabilities of video analysis and contribute to the field of multimedia forensics.

Building upon our previous work [36], the current paper addresses the problem of video-based source camera identification. The decision to pursue a separate study dedicated to video data stems from several factors. Firstly, video data presents unique challenges compared to static images, such as motion, temporal variations, and compression artifacts. These factors require specialized analysis tailored specifically to video-based source camera identification. Secondly, the dataset used for this analysis is inherently different from our earlier work [36], necessitating the adoption of different comparison techniques to ensure accurate identification. Given the complexity and distinct characteristics of video data, it warrants a comprehensive investigation and analysis in its own right. By conducting a separate study focused solely on video-based source camera identification, we can explore the intricacies of video data, assess the performance of our previous methodology, and adapt it accordingly to address the challenges specific to videos. This ensures a thorough understanding of the capabilities and limitations of our approach when applied to video content, while also allowing for fair comparisons with existing methods developed for video-based source camera identification.

The proposed work makes following significant contributions: (i) We introduce an innovative method for video-based source camera identification, building upon the image-based technique. This approach harnesses a new device-specific fingerprint obtained through a data-driven system. (ii) Our system’s efficacy stems from the unique fingerprint extracted from the low- and mid-frequency bands. This fingerprint’s resilience to in-camera processing effects and distortions arising during video processing enhances its robustness, overcoming limitations of high-frequency-based methods vulnerable to noise, compression, and processing operations. (iii) By leveraging camera-specific information within the low and mid-frequency bands, our system accurately identifies source cameras for both images and videos. This breakthrough unifies source identification, addressing a critical barrier in multimedia forensics. It eliminates the need for separate methodologies, allowing seamless identification across various media types. (iv) The extensive experimentation on a recent smartphone benchmark dataset demonstrates consistent superiority over state-of-the-art methods. Our proposed system sets a new benchmark for both source camera model and individual device identification.

The remainder of the paper is structured as follows. Section 2 provides a review of related works. Section 3 presents the proposed data-driven approach to explore the nature of the new fingerprint in the context of video-based source camera identification. Section 4 presents the experimental results and discussions based on our comprehensive evaluation. Finally, Section 5 concludes the paper by summarizing the key findings and their implications for future research in this area.

## 2. Literature Review

Though several successful efforts have been made to identify the source of images, significantly less study has been done to date to address the challenge of video-based source camera identification. In part, this is because of the lack of standard digital video datasets, which are required to build and assess state-of-the-art video forensic techniques. To address this need, Hosler et al. [42] developed a large-scale video forensics database that consists of over 12,000 videos captured using 46 devices of 36 camera models. More recently, Akbari et al. [43] created a video dataset named Qatar University Forensic Video Database (QUFVD). The dataset includes 6000 videos captured with 20 modern smartphones. Despite the capacity of a digital camera to capture both images and videos, the applicability of existing techniques for identifying the camera model of an image is not directly transferable to video camera model identification, as indicated by a recent study [44]. To substantiate this claim, Hosler et al. [44] conducted an experiment employing a CNN originally devised by [45] for image-based source identification. In their research, CNN exhibited remarkable accuracy of 98% when identifying the camera model from a set of images taken from 10 distinct camera models. However, when this same system was tested on videos captured by the same camera model, the identification accuracy significantly dropped to an unacceptable level of 7.5%. To address this challenge, Hosler et al. [44] developed a novel CNN system specifically designed to analyze small patches extracted from video frames, aiming to improve the accuracy of camera model identification. The CNN generated forensic information for each patch, which was subsequently fed into a fusion system. This fusion system employed a voting function to aggregate the forensic information and produce a single, more precise video-based source identification result. Remarkably, this approach achieved a significantly enhanced accuracy rate of 95.8%. Mayer et al. [46] developed a deep learning-based system that uses a CNN-based feature extractor and a similarity network to address the significant limitation of [44], referred to as the open-set scenario. Iuliani et al. [47] investigated a new approach that uses images and videos synergistically to evaluate the device from which they both originate. In particular, the work proved it possible to identify the source of a video by exploiting the reference sensor pattern noise estimated from images captured by the same device. Also, the work showed that the strategy is effective even with digitally stabilized videos. Kouokam and Dirik [48] proposed a pioneering method to estimate the PRNU fingerprint from video frames, specifically addressing the influence of video compression on PRNU noise for source identification purposes, particularly in the context of YouTube videos. Their research aimed to explore the impact of heavily compressed videos and video motion on the performance of source camera attribution. The performance of the camera attribution technique experienced a noticeable reduction due to these factors.

Given the recent advancements in video stabilization, the robustness of PRNU-based techniques is strongly hindered. The approach proposed by Mandelli et al. [40] to deal with video stabilization focussed on extracting reference pattern noise from weakly stabilized videos. The experiments provided insight into the effect of video stabilization employed by recent smartphones on video frames. Altinisik and Sencar [39] developed a source camera verification method for strongly stabilized videos by taking into consideration the spatially variant nature of stabilization transformations. The evaluation of various datasets shows that the method is able to verify the source of 23–30% of all videos that suffered stronger stabilization. However, the method suffers from false PRNU matching due to content similarity among frames. Montibeller et al. [49] proposed to reduce the computation burden in estimating the PRNU from stabilized videos by using the parallelization capabilities of Graphics Processing Units (GPUs).

Akbari et al. [43] conducted a study focused on the development of a dataset called QUFVD and evaluated the performance of source camera identification using a CNN presented in [50]. The evaluation encompassed two distinct scenarios: one involving 10 different camera models to address the task of source camera model identification, and another involving 20 cameras (10 different camera models, each with two devices), to address individual device identification within the context of videos. In the camera model identification scenario, the CNN achieved a notable identification accuracy of 78.8%. This outcome indicates that the CNN-based approach was effective in accurately determining the camera model used to capture the video footage. However, in the task of individual device identification, the performance of the CNN exhibited a relatively lower accuracy rate of 51.2%. This result indicates that distinguishing between individual devices within a camera model based solely on video content proved to be a more challenging task. Akbari et al. [51] improved the source identification accuracy by integrating a PRNU-based layer into their CNN, thereby harnessing the strengths of both PRNU and machine learning methods. By combining the capabilities of both PRNU and machine learning techniques, they developed a novel CNN architecture called PRNU-net. The PRNU-net was specifically designed to address the task of source camera model identification. The experimental evaluation conducted by the authors demonstrated promising results, with the PRNU-net achieving an identification accuracy of 85.7% across 10 different camera models. Bennabhaktula et al. [52] explored the relationship between network complexity and performance in source camera identification by employing CNNs with increasing levels of sophistication. The aim was to investigate whether certain components, such as constrained convolutional layers and residual noise images, were essential for accurate identification. Through a series of experiments, the researchers discovered that sophisticated CNN architectures, such as MobileNet, did not require constrained convolutional layers or residual noise images to achieve high performance in source camera identification. In fact, the results indicated that these additional components were unnecessary when utilizing advanced CNN models. In the context of the QUVFD dataset and the specific task of individual device identification using 20 devices, the MobileNet architecture, even without any constrained convolution, achieved an impressive identification accuracy of 71.75%.

Despite numerous attempts made in recent years to achieve video-based source identification, these efforts have been hindered by the disruptive effects caused by video generation processes like stabilization and video coding. As a result, the existing techniques for mapping images to the exact camera of origin have not proven to be forensically satisfactory, particularly in scenarios involving multiple devices of the same model. However, our research has demonstrated the potential of a novel device-specific fingerprint, as observed in our previous seminal work [36], which exhibits advantageous characteristics such as stochasticity and location independence. Based on these findings, we hypothesize that the robustness of this new fingerprinting approach could be extended to video-based source camera identification, an area that presents additional challenges in achieving accurate identification. To investigate this hypothesis, our objective is to assess the feasibility and performance of the new device fingerprint within the context of video analysis. By conducting a comprehensive evaluation, we aim to determine if this fingerprinting technique can overcome the existing limitations and provide a more reliable method for attributing videos to their respective source cameras.

## 3. Methodology

**Figure 1:**
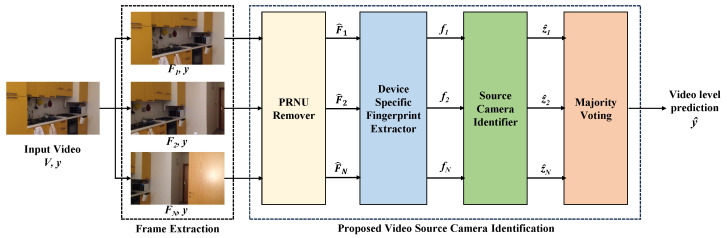
Overview of the proposed methodology.

In this work, we extend the methodology built upon our previous work [36] to video-based source camera identification. In Figure 1, we provide an overview of our methodology, which encompasses the following essential steps: (i) extraction of frames from the input video, enabling us to work with individual frames as the basic unit for subsequent processing and analysis, (ii) PRNU removal process to ensure that our analysis is not influenced by the PRNU fingerprint, (iii) device fingerprint extractor which utilizes CNN to extract distinctive features from the frames, capturing the inherent characteristics of the camera’s imaging system to create a device-specific fingerprint, (iv) source camera identifier, which uses machine learning classifier to perform frame-level source identification; and (v) majority voting, which aggregates the frame classifications to produce video-level classifications. In the following subsections, we will delve into each module, providing the architecture details and specific techniques employed in each step.

### 3.1. Frame Extraction

The first step involves extracting frames from the input video which serve as the input data for subsequent processing and analysis. To achieve this, we begin by converting videos (V) into frames $\left(F=F_{1},F_{2},\cdots F_{N}\right)$, specifically focusing on extracting I-frames. I-frames, also known as keyframes, are essential in video compression and encoding. They are fully encoded frames that do not depend on any other frame for decoding, making them suitable for independent analysis. By selecting I-frames from videos for our analysis, we ensure that we have representative frames that capture the essential information required for source camera identification. This approach allows us to focus on the key frames while reducing the computational complexity associated with processing the entire video sequence. Moreover, previous studies [39,42,52] on video-based source identification have highlighted the effectiveness of utilizing I-frames for camera model identification. To accomplish this, we utilize the *FFmpeg* library in Python to extract I-frames from the videos.

### 3.2. PRNU Remover

The methodology employed in this study is built upon our previous work [36], where we successfully eliminated the PRNU fingerprint from images using two procedures: down-sampling and random-sampling. The first procedure, PRNU removal through down-sampling, involves down-sizing the I-frames to the input size of the proposed CNN architecture using a bilinear filter. By reducing the dimensions of the frames, we ensure that the PRNU fingerprint is effectively eliminated. To evaluate the effectiveness of PRNU removal through down-sampling, we calculate the Peak-to-Correlation Energy (PCE) between the noise residual extracted from the test frames and the reference PRNU of the corresponding source camera by incorporating the process outlined in our previous work [36]. The second procedure, random-sampling, involves the creation of patches by selecting pixels from random locations within an I-frame. These randomly sampled pixels are then used to generate patches of the same size as the input size of the CNN architecture.

**Figure 2:**
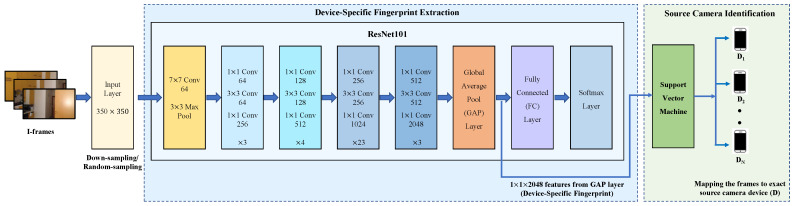
The hybrid ResNet-SVM system to extract the new device fingerprint from video frames to perform frame-level source camera identification.

The PRNU removal process acts as the *input layer* for the device fingerprint extractor (as shown on the left-hand side in Figure 2), which utilizes a CNN architecture developed in our previous work [36]. By formulating the PRNU removal process as the *input layer*, we ensure that subsequent analysis performed by the device fingerprint extractor is independent of the PRNU fingerprint. The down-sampling and random-sampling technique applied to the frames aligns them with the input size requirements of the proposed CNN architecture, specifically the residual network. By applying down-sampling and random-sampling techniques to the I-frames in the *input layer*, we effectively eliminate the PRNU fingerprint while preserving the device-specific fingerprint in low- and mid-frequency bands of images as described in [36] for accurate source camera identification. These procedures serve as key steps in preparing the video frames for subsequent analysis using the proposed CNN architecture.

### 3.3. Device-Specific Fingerprint Extractor and Source Camera Identifier

Building upon the insights mentioned earlier, we leverage our previously developed CNN architecture, described in [36], to carry out video-based source camera identification. In our approach, we specifically employ the ResNet101, a residual neural network with 101 layers (as illustrated in Figure 2). This choice is motivated by the demonstrated effectiveness of ResNet101 in extracting the non-PRNU fingerprint, as showcased in our previous work [36]. However, to adapt the network for video analysis, we introduce several modifications to enhance its performance and robustness. These modifications aim to capture the unique characteristics of the video data. The details of these modifications will be discussed in detail below.

One of the key modifications involves adjusting the input size of the ResNet101 system. In our earlier work [36], the default input size of 224 × 224 pixels was used for image-based experiments. However, the size of the frames can impact CNN’s performance. To address this consideration, we made a modification to the *input layer* of our ResNet101 system. Therefore, we down-sample/random-sample the frames in the *input layer* to 350 × 350 pixels. This particular size has been shown to yield improved performance in the literature [43]. The rationale behind this modification lies in the desire to capture more detailed information from the frames, which may enhance the discriminative capabilities of CNN. By increasing the input size, we allow the network to extract and analyze finer-grained features from the video frames, potentially leading to improved accuracy in source camera identification. While larger patch sizes offer more information for improved accuracy, they also introduce a higher computational burden. However, we will evaluate the trade-off between accuracy and computational efficiency in Section 4.3. By conducting rigorous experiments and analysis, we will assess the impact of the chosen patch size on the overall performance of our video-based source camera identification system.

Continuing to enhance the model’s performance and robustness, we introduce an additional modification: the incorporation of *L2 regularization* into the loss function. In our previous work [36], we minimized the categorical cross-entropy loss function given in Equation (1) for image-based source identification, where *D* represents the number of cameras used for the evaluation, $\hat{y}$ is the class distribution predicted by ResNet101 and *y* represents the one-hot encoded ground-truth class vector of the PRNU-free input I-frame.

(1) $$L\left(\hat{y},y\right)=-\sum_{i=1}^{D}y_{i}\log\left({\hat{y}}_{i}\right)$$

By adding *L2 regularization*, we introduce a regularization term to Equation (1) that penalizes large weights in the CNN. The addition of *L2 regularization*, represented by Equation (2) serves multiple purposes in the context of video-based source camera identification.

(2) $$L\left(\hat{y},y\right)=-\sum_{i=1}^{D}y_{i}\log\left({\hat{y}}_{i}\right)+\lambda \sum_{i=1}^{D}{∥w_{i}∥}^{2}$$

It mitigates the risk of overfitting by discouraging the CNN from relying heavily on specific features present in the training data but not representative of the entire video dataset. The *L2 regularization* term encourages the weights (w) of the ResNet101 to be close to zero, thereby reducing the model’s complexity and preventing overfitting. This regularization technique promotes better generalization to unseen video samples, thereby improving the model’s ability to accurately identify the source camera. The hyperparameter lambda (λ) controls the strength of the regularization effect. The value of λ needs to be determined empirically, as there is no universal best value that suits all scenarios. The optimal value of λ can vary depending on the dataset and network architecture. Therefore, we empirically find the best value of λ that balances the regularization effect without compromising the ResNet’s ability to accurately identify the source camera. Moreover, *L2 regularization* enhances the CNN’s stability and robustness by reducing the influence of outliers and noisy data points. By assigning lower weights to less informative features, the regularization term makes CNN less sensitive to noise, improving its performance on videos with varying levels of noise or disturbances.

In our previous work [36], it was observed that a 2048-dimensional feature vector extracted from the Global Average Pooling (GAP) layer of ResNet101 serves as the device-specific fingerprint for images. It was found that features extracted from the GAP layer improved the overall performance compared to the features from the last Fully Connected (FC) layer. Building upon this insight, we extend the same settings for video-based source identification. For video analysis, we extract a 2048-dimensional feature vector (f) from the GAP layer of ResNet101 to represent the device fingerprint. These features serve as the informative representation of the video-based source, facilitating the subsequent classification and matching tasks in our video-based source camera identification system.

The next step is to provide evidence that the new device-specific fingerprint, extracted from videos, can effectively identify the source device. To achieve this, we employ an SVM classifier, as described in our previous work [36]. The SVM classifier is specifically used for source camera identification of videos at the frame level, as illustrated on the right-hand side in Figure 2, mapping each I-frame extracted from the videos to the corresponding source camera that captured the video. The device-specific fingerprint, represented by the 2048-dimensional feature vector extracted from PRNU-free I-frames by the GAP layer of ResNet101, serves as the input to the SVM classifier. By training the SVM classifier on fingerprints of I-frames with known source cameras, we enable it to learn the distinctive patterns and characteristics associated with each source camera.

### 3.4. Majority Voting

After performing source camera identification at the frame level using the SVM classifier, the subsequent step is to consolidate the frame-level predictions and make a video-level identification. This is achieved through a majority voting scheme. For each video *V*, the frame-level predictions obtained from the SVM classifier are aggregated. The predicted source camera for each frame is recorded, and the most frequently predicted source camera across all frames is selected as the final video-level identification. To implement this scheme, we begin by creating a set *F* that comprises *N* I-frames extracted from the video *V*, as described in Section 3.1. Subsequently, each input frame $F_{n}$ undergoes processing by the fingerprint extractor. The device-specific fingerprint $f_{n}$ that is extracted is then fed into the SVM classifier, resulting in the class prediction ${\hat{z}}_{n}$ for the frame $F_{n}$. The prediction ${\hat{z}}_{n}$ represents a camera device d∈D, where *D* denotes the set of candidate devices utilized for evaluation. To derive the predicted label ${\hat{y}}_{v}$ for the input video *V*, we apply a majority voting procedure to the frame-level predictions ${\hat{z}}_{n}$, with n∈[1,N]. The majority voting scheme ensures that the final identification is based on the collective decision of the individual frame predictions. By considering the majority prediction, we reduce the influence of potential outliers or misclassifications at the frame level, leading to a more reliable and robust video-level identification.

## 4. Experiments and Discussion

### 4.1. Datasets

To assess the effectiveness of the novel device fingerprinting method in the domain of video analysis, specifically video-based source camera identification, we conducted experiments using two distinct datasets: the QUFVD [43] and the VISION dataset [53]. The QUFVD comprises videos captured by 20 recent smartphone cameras. This dataset facilitates device-level identification in the context of video analysis, as it encompasses 10 different camera models, with each model represented by two devices. Additionally, this dataset provides access to I-frames extracted from each video, with a total of 76,531 I-frames. The inclusion of videos from recent smartphone models is particularly advantageous as it enables us to evaluate the proposed system’s performance in the presence of sophisticated in-camera processing commonly employed by modern cameras.

The VISION dataset [53] consists of both images and videos captured by the same camera, allowing us to investigate the performance variation of the new device-specific fingerprinting technique across different media types. This dataset encompasses 35 cameras, from which we selected 15 cameras for evaluation based on specific criteria. Firstly, we considered the number of images available for each camera, prioritizing those with a minimum count of 200 images. This criterion ensures an adequate amount of data for training and testing purposes. Additionally, to prevent CNN predictions from being influenced by similar scene content, we discarded cameras that exhibited high similarity between images in the training and testing sets for the same camera. This precaution ensures that any observed performance improvements are not attributable to scene content but rather to the intrinsic fingerprinting characteristics. By adhering to these criteria, we identified and included the following 15 cameras for our evaluation: D01-Samsung Galaxy S3 Mini, D03-Huawei P9, D04-LG D290, D11-Samsung Galaxy S3, D12-Sony Xperia Z1 Compact, D15-iPhone 6, D16-Huawei P9 Lite, D28-Huawei P8, D30-Huawei Honor 5c, D31-Samsung Galaxy S4 Mini, D34-iPhone 5, two devices of iPhone 5c (D14 and D18), and two devices of iPhone 4s (D02 and D10). For video-based source identification, we extract I-frames from videos captured with these 15 cameras using the *FFmpeg* library in Python, which results in 11,244 and 9833 I-frames for training and testing, respectively. By employing these two datasets and their associated criteria for camera selection, we aim to provide a comprehensive and unbiased evaluation of the new device-specific fingerprinting approach for video-based source camera identification.

### 4.2. Validating PRNU Removal

To validate the effectiveness of PRNU removal, we employ the following procedure for each camera in the QUFVD [43]. Initially, we generate the reference PRNU fingerprint by extracting noise residuals from 50 I-frames within the training set. Subsequently, we extract noise residuals from 30 original I-frames within the test set for each camera. The similarity between the reference PRNU and the noise residuals extracted from the 30 original I-frames is evaluated using the PCE metric. To assess the efficacy of PRNU removal, we apply down-sampling to the same test I-frames and measure the PCE between the noise residuals extracted from the 30 down-sampled I-frames (PRNU-free I-frames) and the reference PRNU of the corresponding source camera. We perform a similar evaluation procedure for the cameras in the VISION dataset. In this case, we extend the analysis to include both images and I-frames extracted from videos within the VISION dataset. To quantitatively assess the effectiveness of PRNU removal, we present the PCE values before and after PRNU removal in Table 1 for a subset of cameras. The PCE values indicate the degree of similarity between the noise residuals and the reference PRNU fingerprint.

For the VISION dataset, particularly for images, the average PCE values for the original noise residuals were observed to be very high. However, after PRNU removal, there was a significant reduction in PCE values, which fell below the decision threshold of 50 [3], demonstrating effective PRNU removal for images. In the case of videos, both in the VISION and QUFVD datasets, the PCE values for the original noise residuals were already considerably lower than the decision threshold of 50. This indicates that the video processing techniques incorporated by the cameras during video generation have a disruptive effect on the intrinsic PRNU fingerprint, suggesting that the PRNU fingerprint is not as reliable in videos due to the impact of video processing techniques. For example, in the QUFVD dataset, the “Huawei Y7–1” camera exhibits an average PCE of 3.1022 for the original noise residuals, which further decreases to 1.4756 after PRNU removal. This significant reduction in PCE indicates the successful removal of PRNU noise.

### 4.3. Building the Baseline for Video-based Source Camera Identification

In this section, we aim to establish a baseline for video-based source camera identification by leveraging our device-specific fingerprint extractor, source camera identifier, and majority voting, built upon the insights described in Section 3. During training, we trained the ResNet101 architecture for 20 epochs using the Adam optimizer. The mini-batch size was set to 12, and the learning rate was set to 0.0001. All experiments were conducted using MATLAB 2020b. We conduct a series of experiments to evaluate the performance and effectiveness of our approach.

First, we train the device-specific fingerprint extractor, where the default input size of 224 × 224 pixels was used, as described in [36]. This fingerprint extraction process is applied to a diverse set of videos obtained from different camera sources in the QUFVD. By testing our device-specific fingerprint extractor and source camera identifier on this input size, we establish a baseline performance for comparison. This allows us to assess the initial effectiveness of our system and validate the consistency of results with our previous study.

Subsequently, we expand our investigation by down-sizing the frames to 350 × 350 pixels. This modification aims to capture more detailed information from the video frames, potentially improving the discriminative capabilities of our fingerprint extractor. Through rigorous experimentation and analysis, we assess the performance of our system on this larger input size and compare it to the baseline results obtained with the default input size. The results in Table 2 demonstrate the impact of input size on the performance of our video-based source camera identification system in terms of frame-level and video-level identification accuracy.

When using the default input size of 224 × 224 pixels, we achieved an accuracy of 90.39% and 91.67% for frame-level and video-level identification, respectively, in the experiment involving two devices of the Huawei Y7 model. These accuracy metrics serve as the baseline performance for our system, providing a reference for comparison. However, when we increased the input size to 350 × 350 pixels, we observed a significant improvement in identification accuracy. The accuracy increased to 94.26% and 95% for frame-level and video-level identification, respectively, indicating a higher rate of correctly identifying the source camera. Furthermore, as the number of cameras increased, the larger input size demonstrated its advantages in video-based source camera identification. We observed an improvement of over 8 percentage points in identification accuracy for both frame-level and video-level analyses. This result highlights the benefit of using a higher input size, as it allows for capturing more detailed information from the frames and enhancing the overall accuracy of our system. The larger input size provides a richer representation of the device fingerprint, leading to improved accuracy in identifying the source camera.

In addition to investigating the impact of input size on video-based source camera identification, we further enhanced our experimental setup by incorporating *L2 regularization* into the categorical cross-entropy loss function. To evaluate the effectiveness of this modification, we conducted experiments by down-sizing the frames to 350 × 350 pixels, as determined in our previous investigation. By applying *L2 regularization* to the loss function, we introduced a regularization term that encouraged the model to learn more generalizable features and reduce the reliance on specific training samples. The regularization parameter, denoted as λ, controls the strength of the regularization in the loss function and plays a crucial role in balancing model complexity and generalization. We conducted empirical experiments where we trained the feature extractor across a range of λ values, spanning from 0.1 to 0.0001. Our objective was to identify the λ value that would yield optimal results. Through this empirical investigation, we found that a λ value of 0.005, when combined with a learning rate of 0.0001, yielded the most optimal outcomes on the test set. To maintain the focus on essential content and avoid redundancy, we inserted only the best result obtained in this paper. Through experimental evaluation, we quantify the impact of regularization on the performance of our video-based source camera identification system and determine its effectiveness in enhancing the baseline results.

The results obtained from these experiments, summarized in Table 3, reveal notable improvements in the identification accuracy of our video-based source camera identification system. When compared to the baseline performance achieved with the default input size of 224 × 224 pixels and increased input size of 350 × 350 pixels, incorporating *L2 regularization* led to a further increase in accuracy for both frame-level and video-level identification. For example, for the experiment involving 20 devices of the QUVFD, we observed an accuracy of 77.95% and 79.58% for frame-level and video-level identification, respectively. This represents a significant improvement over the baseline accuracy of 71.65% and 73.08% achieved without *L2 regularization*. Therefore, by comparing the results in Table 3 with baseline accuracy in Table 2 for device-level identification (20 devices of the QUFVD), we observed an average improvement of 15 percentage points in identification accuracy for both frame-level and video-level analyses. These findings highlight the efficacy of increased input size and *L2 regularization* in enhancing the generalization capability of our system, leading to improved accuracy in identifying the source camera.

As demonstrated, increasing the input size of the fingerprint extractor result in increased accuracy. However, it is important to ensure that this improvement does not come at the cost of increased computation time. We tested the performance of the trained fingerprint extractor using an HP EliteDesk 800 G4 Workstation equipped with an NVIDIA GeForce GTX 1080 GPU, a 3.7 GHz Intel processor, and 32 GB RAM. The computation time required by the fingerprint extractor trained on different sets of cameras to downsize the frames to specific input sizes and extract the fingerprint is presented in Table 4. From the table, it can be observed that increasing the input size has a minimal effect on the computation time. For instance, the fingerprint extractor trained on two devices of the Huawei Y7 model took 166.1 ms to downsize a single frame to 224 × 224 and extract the fingerprint. Increasing the input size to 350 × 350 pixels resulted in a slight increase in the computation time to 170.3 ms. This marginal increment in the computation time yields a significant improvement in identification accuracy, as shown in Table 2 and Table 3. It is important to strike a balance between accuracy and computation time when determining the optimal input size for the fingerprint extractor. The results indicate that increasing the input size can lead to improved accuracy without a substantial increase in computation time, making it a favorable trade-off for practical applications.

**Figure 3:**
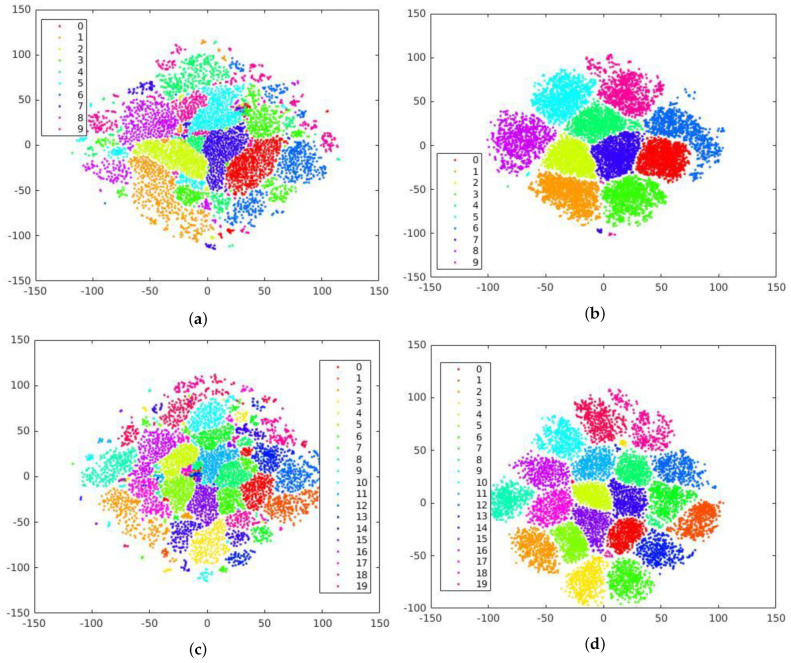
t−SNE Visualization of class separation for 10 different camera models (top) and 20 devices (bottom) of the QUFVD obtained using fingerprint extractor with default settings (224 × 224, without regularization) in (**a**,**c**) and modified settings (350 × 350, with regularization) in (**b**,**d**) demonstrating the enhanced discriminative capability of the proposed system. (The x and y axes represent a two-dimensional space where high-dimensional data points are projected using t-SNE. The positions of data points in this plot are determined by their relationships and similarities in the original high-dimensional space).

Furthermore, we demonstrate the impact of these modifications on the class separation capability of the proposed system by applying t-distributed Stochastic Neighbor Encoding (t-SNE) to the feature vector extracted by the proposed device fingerprint extractor. The t-SNE visualization results depicted in Figure 3 provide valuable insight into the discriminative power of our system, showcasing its ability to capture and represent unique characteristics specific to each device. The visualizations in the first column demonstrate poor class separation when the fingerprint extractor is used with default settings. The features appear randomly distributed, lacking clear boundaries between camera classes. In contrast, the incorporation of increased input size and *L2 regularization* have significantly improved class separation, resulting in distinct clusters of features from the same source, as depicted in the second column. This showcases the enhanced discriminative capability of our proposed system, allowing for more accurate identification of the source camera. These modifications have significantly enhanced the performance of our approach, establishing a strong baseline for further evaluation and analysis of video-based source camera identification.

To comprehensively assess the efficacy of our proposed method, we further carried out a study involving different machine learning classifiers to determine the most suitable framework for source identification. In particular, we compared the performance of SVM, K-Nearest Neighbors (KNN), and neural networks. Through our empirical investigations, we aimed to identify the classifier that achieves the highest classification accuracy when integrated with our ResNet101 feature extractor. Notably, employing SVM in conjunction with ResNet101 consistently demonstrated superior performance compared to the alternative classifiers. In one illustrative experiment involving two Huawei Y7 devices, we achieved an impressive frame-level identification accuracy of 95.02% by employing SVM with ResNet101, utilizing an input size of 350 × 350 pixels. By contrast, KNN yielded an identification accuracy of 85.22%, and the neural network achieved 92.96% accuracy under the same experimental conditions. The discernible advantage of SVM over these alternative classifiers in terms of classification accuracy prompted our strategic decision to incorporate it as a pivotal component in our proposed method. These results highlight the synergistic relationship between our proposed system’s components, ensuring that the accuracy benchmark we have established is obtained by rigorous experimentation and comparison across different machine learning classifiers.

### 4.4. Assessing the Strength of the New Fingerprint

In this sub-section, we aim to assess the strength and significance of our new fingerprint in the field of video-based source identification. To achieve this, we compare the performance of our proposed system with other existing methods [43,51,52] that have demonstrated benchmark results on the QUFVD [43] through two experiments: source camera model identification (SCMI) and individual source camera identification (ISCI). Firstly, in Table 5, we compare the results of the experiment involving 10 different camera models present in the QUFVD dataset to evaluate the performance of our system in SCMI. At the frame-level, it was hard for PRNU-net [51], which combines the benefits of PRNU fingerprint and deep learning, to identify the Huawei Y7 model due to the lower resolution of videos, resulting in a lower performance of 71.5% compared to other models. However, our proposed system achieved higher identification accuracy for the Huawei Y7 and Huawei Y9 models, despite their lower resolution of 720 × 1280 compared to other cameras with 1080 × 1920 resolution. This contrasts with the findings in [43,51] where they reported lower performance for the Huawei Y7 model. In particular, our proposed system, leveraging the new device fingerprint, demonstrated remarkable performance for the Huawei Y7 model, achieving 98.40% accuracy at the frame level and 99.20% accuracy at the video level. Additionally, our system exhibited exceptional performance, attaining a perfect 100% accuracy for the Huawei Y9 model. This indicates the robust capability of our new fingerprint in identifying the source even when the video resolution is low. Overall, our proposed system outperformed the other existing methods in terms of overall identification accuracy at the frame-level and video level.

In [43,51], the authors also faced challenges in identifying cameras such as iPhone 8 Plus, and Redmi Note 9 Pro, resulting in lower performance. However, our proposed system demonstrated robust performance, achieving more than 90% identification accuracy for these challenging camera models. Additionally, among the 10 camera models in the QUFVD, the iPhone XS Max model exhibited lower performance compared to others. Notably, the iPhone XS Max and Samsung Note 9 utilize the H.265 codec, while the other cameras in the dataset use H.264. Despite the Samsung Note 9 achieving a good performance of 93.3% at the video level, the iPhone XS Max showed only 85.80% accuracy. These observations suggest that the codec alone may not have a significant impact on the results, and further investigation and analysis are necessary to uncover the underlying factors influencing the performance on specific camera models, which could be the imaging technique used by the iPhone models.

**Figure 4:**
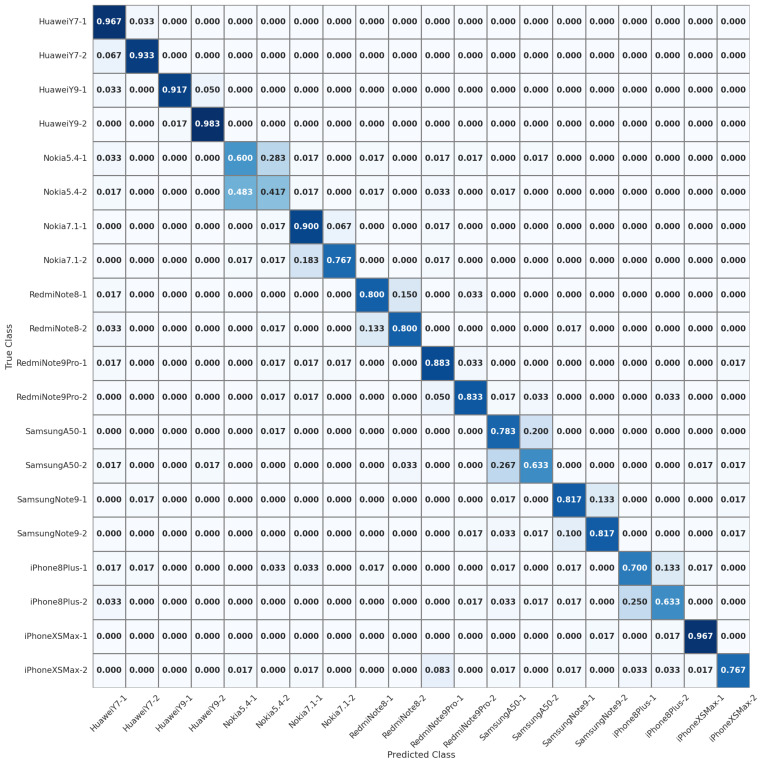
Normalized confusion matrix for ISCI.

Further, we proceed to the experiment of individual source camera identification, where we aim to identify specific devices among a set of 20 devices in the QUFVD dataset. This device-level identification scenario allows us to evaluate the effectiveness of our new fingerprint in distinguishing between different devices. ISCI scenario is more challenging than SCMI. As illustrated by the confusion matrix in Figure 4, misclassification between devices of the same brand occur in most cases. Across multiple camera devices, our method consistently demonstrates improved identification accuracy compared to the state-of-the-art MISLnet [43] and MobileNet [52] methods. To the best of our knowledge, the current benchmark accuracy for ISCI stands at 71.75%, as established by the CNN proposed in [52]. Significantly, as highlighted in Table 6, our system, leveraging the new device-specific fingerprint, surpasses the existing best performance in individual device identification, achieving an identification accuracy of 79.58% in the experiment involving 20 devices from the QUFVD. Furthermore, referring to the ISCI findings in [43], it is evident that the MISLnet method exhibits the lowest performance on certain devices, such as HuaweiY7-1, Nokia5.4 devices, Nokia7.1-2, RedmiNote9Pro devices, SamsungA50-2, and iPhone8Plus devices. For instance, for the Huawei Y7-1 device, our method achieves a video-level accuracy of 96.67%, surpassing the accuracy of 45% achieved by the MISLnet CNN method. Notably, our system demonstrates remarkable improvement, exceeding the performance of MISLnet [43] by more than 40 percentage points on devices where the latter struggled to attain even 40% accuracy, a level significantly lower than random guessing. For instance, while the MISLnet achieved a minimum accuracy of 20% on the second device of the iPhone 8 Plus model, our system achieved a far superior accuracy of 63.33%. This consistent superiority holds true for other devices where the MISLnet achieved the lowest performance.

This comparison highlights the enhanced capabilities of our system and establishes its significance in the field. The improved performance of the proposed system in both SCMI and ISCI can be attributed to the robust characteristics of the new device fingerprint. Unlike other methods that rely on patches cropped from images or the PRNU fingerprint, our system is designed to capture and utilize the low- and mid-frequency bands of the images. The down-sampling operation, achieved by applying a bilinear filter (low-pass filtering), effectively removes the high-frequency components from the video frames. Consequently, during the learning process of the non-PRNU device fingerprint by the ResNet101 system, only the low and mid-frequency components of the frames are considered. This characteristic indicates that the features extracted by the proposed system primarily stem from the low and mid-frequency components of the video frames, as demonstrated in our earlier work [36]. This makes our system resilient to disruptive effects that primarily affect the high-frequency bands and gives our system an advantage in maintaining the intrinsic characteristics necessary for accurate source camera identification. As a result, our system is less susceptible to video processing techniques that may impact the high-frequency bands, ensuring the preservation of essential device-specific information. The improved performance of our proposed system in the context of ISCI, surpassing other state-of-the-art techniques, strongly demonstrates the device-specific characteristics of our new fingerprint. This uniqueness contributes significantly to performance improvement, particularly in identifying individual devices. Our system effectively leveraging these distinct features, allows for more precise and reliable identification compared to other existing techniques.

### 4.5. Confirming Stochasticity

In this subsection, we aim to provide empirical evidence to support our hypothesis that the new device-specific fingerprint, being stochastic, has the potential for video-based source identification. This hypothesis is based on the observation from our previous seminal work [36] on image-based source identification, where experiments conducted on image patches formed with randomly selected pixels revealed the stochastic nature of the new fingerprint. Unlike the deterministic nature of the PRNU fingerprint, which is vulnerable to desynchronization operations and pixel misalignment, our new fingerprint exhibits a stochastic nature that is resilient to such challenges [36]. This characteristic makes it well-suited for video-based source identification, as videos often undergo stabilization processes that may introduce pixel misalignment during the capturing process.

To validate this hypothesis, we conduct experiments on patches formed by randomly sampling pixels from the I-frames in the QUFVD and VISION datasets to further confirm the stochastic nature of the new fingerprint. For each I-frame, we create a patch of size 350 × 350 by selecting pixels from random locations within the frame. This random sampling disrupts the structural relationship between neighboring pixels and effectively destroys the PRNU fingerprint. By evaluating the performance of our proposed system on these randomly sampled patches, we can demonstrate that the achieved accuracy is indeed based on the new fingerprint and not attributed to the PRNU fingerprint. The results of these experiments are presented in Table 7. The system achieves competitive identification accuracy, particularly for specific device identification in the QUFVD (such as two devices of the Huawei Y7 model) and for the iPhone devices in the VISION dataset. However, as the number of cameras with different models increases, the identification accuracy tends to decrease. This observation highlights the influence of varying camera characteristics and imaging techniques on the identification process. The results confirm that the proposed system can successfully identify the source camera even after disrupting the structural relationship between neighboring pixels. This reaffirms the stochastic nature of the new fingerprint, which serves as a reliable basis for accurate source camera identification. The stochastic characteristics of the new fingerprint play a crucial role in the superior performance of our proposed method compared to existing techniques (as demonstrated in Table 5 and Table 6) that rely on source information in the high-frequency bands, such as the deterministic PRNU fingerprint. Despite the presence of pixel misalignment and other disruptive factors that are common in videos, the proposed system leverages the stochastic characteristics of the fingerprint in the low- and mid-frequency bands to effectively identify the source camera. This emphasizes the robustness of the new fingerprint and its ability to withstand challenging conditions.

### 4.6. Investigation of Fingerprint Consistency

The new device-specific fingerprint has exhibited remarkable performance in both image-based [36] and video-based source identification, as highlighted in the previous sub-sections. In this sub-section, we delve deeper into the characteristics of the new fingerprint by investigating its consistency across different media types. Specifically, we perform source camera identification on images and videos captured from the same camera to analyze how the new fingerprint varies in these distinct formats. To conduct this investigation, we leverage the VISION dataset, which encompasses images and videos acquired using the same camera. This analysis further enhances our understanding of the fingerprint’s behavior and its suitability for source camera identification in various scenarios.

**Figure 5:**
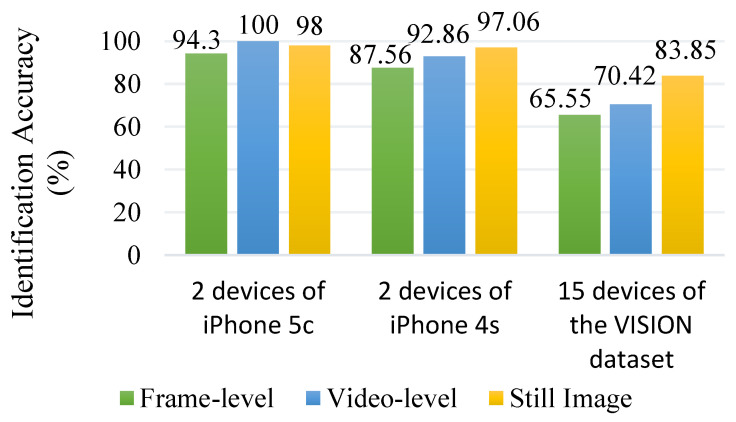
Consistency of the new device fingerprint across different media types.

The results of the source camera identification experiment using down-sampled images and down-sampled I-frames are presented in Figure 5. The identification accuracy is evaluated at the frame-level, video-level, and for still images captured by the same cameras. Notably, high identification accuracy is observed for both still images and videos taken from the same camera, specifically for the two devices of the iPhone 4s model and two devices of the iPhone 5c model. This suggests that the distinctive characteristics captured by the proposed system remain consistent and reliable, regardless of whether it is extracted from images or videos. The fact that the new fingerprint behaves similarly for both images and videos imply that it is not heavily influenced by the specific characteristics of a particular media format. This suggests that the fingerprint’s features are intrinsic to the camera’s imaging system and are independent of the temporal nature of videos or the static nature of still images. This stability is crucial for ensuring accurate and consistent source camera identification, regardless of whether the input is a still image or a video frame.

Our system’s ability to work effectively for both images and videos can be attributed to the nature of the new fingerprint on which our system operates. This fingerprint is extracted from the low- and mid-frequency bands of the images and videos, making it resilient to the disruptive effects caused by sophisticated in-camera processing techniques applied during image and video capturing. The stochastic nature of the new fingerprint further enhances its robustness, allowing it to withstand the challenges posed by various processing techniques, pixel misalignment, and other distortions introduced during video stabilization. By operating on the low- and mid-frequency bands and utilizing the stochastic nature of the new fingerprint, our system overcomes the limitations faced by other existing techniques that rely on high-frequency bands. These limitations often arise from the vulnerability of high-frequency information to noise, compression, and other image/video processing operations. Our system’s robustness to such disruptions allows for accurate source camera identification even when the input media undergoes different transformations or modifications.

The capability of the proposed work is of paramount importance as it can greatly enhance traceability and attribution in real-world forensic situations. However, in some cases, there is a chance that the forensic analyst will not have physical access to the suspect camera responsible for capturing the image under investigation. Our proposed data-driven system, however, needs access to the camera to collect sample images/videos and extract the device fingerprints for effectively identifying the source camera. Therefore, the scope of this research is constrained to exact source camera identification within the context of forensic analysis requiring physical access to the candidate device. Furthermore, an avenue for advancing video-based source identification involves assessing the system’s resilience against disruptive factors like unknown compression artifacts introduced by social network platforms. Given that the new fingerprint predominantly resides in the low and mid-frequency bands, its anticipated robustness against these disruptions that primarily affect the high-frequency band is noteworthy. Exploring how such artifacts influence accuracy and robustness will likely contribute to optimizing the system’s performance in real-world scenarios where videos undergo diverse transformations. Another notable limitation to consider involves potential deliberate interference with the frequency signal in an attempt to disguise camera identification. This concern arises from the fact that malicious actors could manipulate the device-specific fingerprint by intentionally introducing alterations to the low- and mid-frequency bands. While our method is designed to robustly capture the unique characteristics of these frequency components, it’s important to acknowledge the possibility of such deliberate tampering. The challenge lies in developing countermeasures to mitigate the impact of such interference, ensuring the reliability and accuracy of source camera identification even in the face of adversarial attempts to obfuscate the fingerprint. Addressing this potential limitation presents an intriguing avenue for further research, contributing to the development of more resilient forensic techniques.

Our proposed system, which leverages camera-specific information in the low and mid-frequency bands, demonstrates the capability to accurately identify the source camera of both images and videos. This is a significant breakthrough in the field of multimedia forensics, as existing literature suggests that source identification techniques designed specifically for images may not be suitable for videos and often fail to accurately identify the source camera of videos [44]. By utilizing a single framework that can handle both images and videos, we have overcome a significant barrier in multimedia forensics. This unified framework allows for seamless source camera identification across different media types, eliminating the need for separate techniques or methodologies for image and video analysis.

## 5. Conclusions

In this paper, we have introduced a pioneering approach to video-based source camera identification, extending the foundations of image-based technique. This innovation leverages a novel device-specific fingerprint, obtained through a data-driven system, to enhance identification accuracy and robustness. By harnessing the unique fingerprint residing in the low- and mid-frequency bands, our system effectively addresses challenges posed by in-camera and video processing effects, setting it apart from traditional high-frequency-based methods. The proposed system’s ability to accurately identify source cameras in both images and videos unifies source identification, eliminating the need for separate methodologies across different media types. Through comprehensive experimentation on a benchmark dataset of recent smartphones, our proposed system consistently outperforms state-of-the-art techniques, establishing new benchmarks for both source camera model and individual device identification. Our contributions hold great potential for real-world forensic applications, enhancing the accuracy and reliability of source camera identification. In essence, our work propels the field of multimedia forensics towards a future where identification accuracy, robustness, and efficiency converge seamlessly, further enabling investigations across a wide range of multimedia sources and applications.

## Figures and Tables

**Table 1:** Comparison of PCE values before and after PRNU removal.

Camera	Average PCE	
	**Original**	**PRNU-Free**
QUFVD (Videos)		
Huawei Y7–1	3.1022	1.4756
Huawei Y7–2	7.7807	2.0125
Huawei Y9–1	4.8255	1.3045
Huawei Y9–2	7.3392	1.1901
iPhone 8 Plus–1	51.1895	2.8016
iPhone 8 Plus–2	13.9599	2.0407
Samsung A50–1	6.7134	0.9908
VISION dataset (Videos)		
iPhone 5c–1	27.1923	2.5305
iPhone 5c–2	9.7684	1.8854
iPhone 4s–1	2.7800	0.5143
VISION dataset (Images)		
iPhone 5c–1	16,067	2.9315
iPhone 5c–2	9,848	3.2315
iPhone 4s–1	17,674	2.6709

**Table 2:** Evaluation of the impact of input size on proposed video-based source camera identification (Best results are highlighted in bold).

Cameras Used	Identification Accuracy (%)			
	**Frame-Level**		**Video-Level**	
	**224 × 224**	**350 × 350**	**224 × 224**	**350 × 350**
2 devices of Huawei Y7	90.39	**94.26**	91.67	**95.00**
4 devices of the Huawei brand	90.07	**93.41**	91.25	**93.75**
10 Camera Models of QUFVD	83.39	**91.79**	84.42	**92.67**
20 devices of QUFVD	63.06	**71.65**	64.17	**73.08**

**Table 3:** Evaluation of the impact of regularization on proposed video-based source camera identification (Best results are highlighted in bold).

Cameras Used	Identification Accuracy (%)			
	**Frame-Level**		**Video-Level**	
	**Without Regularization**	**With Regularization**	**Without Regularization**	**With Regularization**
2 devices of Huawei Y7	94.26	**95.02**	95.00	**95.83**
4 devices of the Huawei brand	93.41	**93.84**	93.75	**94.75**
10 Camera Models of QUFVD	91.79	**93.67**	92.67	**94.50**
20 devices of QUFVD	71.65	**77.95**	73.08	**79.58**

**Table 4:** Computation time required by the fingerprint extractor at different input sizes.

Trained Fingerprint Extractor	Time Taken to Extract the Fingerprint from Each Frame in Milliseconds (ms)	
	**224 × 224**	**350 × 350**
2 devices of Huawei Y7	166.1	170.3
SCMI	168.2	170.5
ISCI	169.1	172.2

**Table 5:** Performance comparison with other methods for SCMI (Best results are highlighted in bold).

Camera Model	Identification Accuracy (%)					
	**Frame-Level**			**Video-Level**		
	**MISLnet [43]**	**PRNU-Net [51]**	**Our Method**	**MISLnet [43]**	**PRNU-Net [51]**	**Our Method**
Huawei Y7	70.4	71.5	**98.4**	75.8	86.0	**99.2**
Huawei Y9	76.9	77.3	**99.3**	90.8	91.6	**100.0**
Nokia 5.4	81.6	83.1	**90.3**	91.7	**92.7**	90.8
Nokia 7.1	76.7	80.6	**96.6**	79.8	92.2	**96.7**
Redmi Note 8	72.8	81.9	**95.2**	84.2	84.2	**95.0**
Redmi Note 9 Pro	65.0	75.0	**91.7**	70.0	77.3	**93.3**
Samsung A50	68.8	75.0	**96.8**	70.0	77.2	**96.7**
Samsung Note 9	72.0	78.8	**92.4**	87.6	**95.8**	93.3
iPhone 8 Plus	69.6	73.9	**91.2**	68.3	85.5	**94.2**
iPhone XS Max	69.3	79.9	**84.7**	70.0	74.9	**85.8**
Average Accuracy	72.5	77.7	**93.6**	78.8	85.74	**94.5**

**Table 6:** Performance comparison with other methods for ISCI (Best results are highlighted in bold).

Method	Identification Accuracy (%)	
	**Frame-Level**	**Video-Level**
MISLnet-grayscale [43]	49.9	59.6
MISLnet-color [43]	47.9	51.2
MobileNet [52]	-	71.75
Our method	**77.95**	**79.58**

**Table 7:** Evaluation of the stochastic nature of the new fingerprint.

Camera Used	Identification Accuracy (%)	
	**Frame-Level**	**Video-Level**
QUFVD [43]		
2 devices of Huawei Y7	81.69	85.83
SCMI (10 models)	67.48	70.83
ISCI (20 devices)	53.07	56.67
VISION [53]		
2 devices of iPhone 5c	83.86	94.44
2 devices of iPhone 4s	75.05	80.00
15 devices of the VISION dataset	55.53	59.19

## Data Availability

Publicly available datasets were analyzed in this study. This data can be found here: https://lesc.dinfo.unifi.it/VISION/ (accessed on 14 April 2023), https://www.dropbox.com/sh/nb543na9qq0wlaz/AAAc5N8ecjawk2KlVF8kfkrya?dl=0 (accessed on 14 April 2023).
